# New agreement under C-TAP aims to improve global access to COVID-19 testing technologies

**Published:** 2022-07

**16 June 2022 -** A new, open, transparent sublicence agreement between the Medicines Patent Pool (MPP) on behalf of C-TAP, and South African pharmaceutical company Biotech Africa will accelerate the manufacture and sale of a COVID-19 serological antibody technology around the world.

The World Health Organization welcomes the sublicence agreement, the first of its kind to be signed under the auspices of the WHO’s COVID-19 Technology Access Pool (C-TAP) initiative. C-TAP was set up in 2020 to facilitate the timely, equitable and affordable access to COVID-19 health products.

The new agreement builds on a licensing agreement announced by WHO and MPP last year with Spain’s National Research Council (CSIC). The non-exclusive sublicence allows Biotech to manufacture and commercialize CSIC’s COVID-19 serological test worldwide.

“The most effective way to get – and keep - ahead of COVID-19 is to keep testing,” said Dr Tedros Adhanom Ghebreyesus, WHO Director-General. “This new agreement means we can take advantage of untapped manufacturing capacity so more people in more countries can have easier access to affordable diagnostics.”

The test effectively checks for the presence of anti-SARS-CoV-2 antibodies developed either in response to a COVID-19 infection or to a vaccine. The agreement covers all related patents and the biological material needed to manufacture the test. CSIC will provide all know-how to Biotech as well as training. The licence will be royalty-free for low- and middle-income countries and will remain valid until the date the last patent expires.

“BioTech Africa is honoured to have been selected to be the first biotechnology company in Africa to collaborate with C-TAP in order to facilitate the local manufacture of affordable COVID-19 surveillance devices,” said Jenny Leslie, Biotech Africa Chief Operations Officer.

“This recognition is a product of our perseverance and dedication to become a global player in the manufacture of the highest quality recombinant proteins. The signing of this agreement emphasizes the company’s ongoing goal to support diagnostics needs around the world,” Leslie said.

“We are thrilled to see the COVID-19 Technology Access Pool initiative bearing fruits with the goal of providing equitable access to life-saving health products for the world´s most vulnerable people,” said Charles Gore, MPP Executive Director.

Launched in 2020 by the WHO Director-General and the President of Costa Rica, and supported by 44 WHO Member States, C-TAP aims to facilitate timely, equitable and affordable access to COVID-19 health products by boosting their production and supply through open, non-exclusive licensing agreements.

The C-TAP platform provides a global one-stop-shop for developers of COVID-19 therapeutics, diagnostics, vaccines and other priority health technologies to share knowledge and data and license their intellectual property to additional manufacturers through public health-driven, voluntary, non-exclusive and transparent licences.

By pooling technologies, developers of COVID-19 health products can boost manufacturing capacity in all regions and expand access to life-saving tools.

Available from: https://www.who.int/news/item/16-06-2022-new-agreement-under-c-tap-aims-to-improve-global-access-to-covid-19-testing-technologies

